# Carboxydotrophy potential of uncultivated Hydrothermarchaeota from the subseafloor crustal biosphere

**DOI:** 10.1038/s41396-019-0352-9

**Published:** 2019-02-07

**Authors:** Stephanie A. Carr, Sean P. Jungbluth, Emiley A. Eloe-Fadrosh, Ramunas Stepanauskas, Tanja Woyke, Michael S. Rappé, Beth N. Orcutt

**Affiliations:** 1 Bigelow Laboratory for Ocean Sciences, 60 Bigelow Drive, East Boothbay, ME 04544 USA; 2 Hartwick College, Oneonta, NY USA; 3 Center for Dark Energy Biosphere Investigations, University of Southern California, Los Angeles, CA USA; 4 Department of Energy, Joint Genome Institute, Walnut Creek, CA USA; 5 Hawaii Institute of Marine Biology, University of Hawaii at Manoa, PO BOX 1346, Kaneohe, HI 96744 USA

**Keywords:** Biodiversity, Water microbiology, Biogeochemistry

## Abstract

The exploration of Earth’s terrestrial subsurface biosphere has led to the discovery of several new archaeal lineages of evolutionary significance. Similarly, the deep subseafloor crustal biosphere also harbors many unique, uncultured archaeal taxa, including those belonging to *Candidatus* Hydrothermarchaeota, formerly known as Marine Benthic Group-E. Recently, Hydrothermarchaeota was identified as an abundant lineage of Juan de Fuca Ridge flank crustal fluids, suggesting its adaptation to this extreme environment. Through the investigation of single-cell and metagenome-assembled genomes, we provide insight into the lineage’s evolutionary history and metabolic potential. Phylogenomic analysis reveals the Hydrothermarchaeota to be an early-branching archaeal phylum, branching between the superphylum DPANN, Euryarchaeota, and Asgard lineages. Hydrothermarchaeota genomes suggest a potential for dissimilative and assimilative carbon monoxide oxidation (carboxydotrophy), as well as sulfate and nitrate reduction. There is also a prevalence of chemotaxis and motility genes, indicating adaptive strategies for this nutrient-limited fluid-rock environment. These findings provide the first genomic interpretations of the Hydrothermarchaeota phylum and highlight the anoxic, hot, deep marine crustal biosphere as an important habitat for understanding the evolution of early life.

## Introduction

Over the past several years, the advancement of culture-independent techniques has prompted the discovery and genomic characterization of several new archaeal lineages (see refs. [1, 2] and references within). Each new phylum fills gaps in the genomic tree of life, allowing for the continuous reevaluation of the evolutionary models that lead from a common ancestor [2–5] to the splitting of the domains [6–9]. Similarly, analyzing genome annotations of uncultivated lineages has provided valuable insight into each groups’ metabolic potential and prospective geochemical role within their environment [10–12].

Recently, the first genomes from a unique, uncultivated lineage of Archaea, known as *Candidatus* Hydrothermarchaeota (previously Marine Benthic Group-E or MBG-E [13, 14]), were documented through metagenomic sequencing of crustal fluids collected from the deep subseafloor environment of the Juan de Fuca Ridge flank (JdFR; Figure S1) [15]. Samples were acquired using subseafloor borehole observatories called CORKs for (Circulation Obviation Retrofit Kits) that were installed during Integrated Ocean Drilling Program (IODP) Expedition 327 and provide access to the oceanic crust and the fluids circulating therein [16]. In the JdFR environment, fluid circulating between outcrops undergoes extensive fluid-rock reactions [17–19], becoming warm (64 °C), depleted in oxygen and nitrate, and enriched in dissolved metals and reduced gases [19, 20]. Eventually, the chemically altered fluids escape from discharge outcrops and hydrothermal vents, connecting both abiotic and biologically mediated water-rock reactions to global biogeochemical cycles [21, 22].

The fluid circulating through the JdFR basement environment harbors microbial cell densities in the order of 10^4^ cells per ml [23, 24]. The Hydrothermarchaeota appear particularly abundant in the JdFR environment, comprising up to half of the archaeal 16S ribosomal RNA (rRNA) gene amplicons and one-third of the single-amplified genomes (SAGs) sorted from the total community [23]. These abundances are significantly greater than those observed from sedimentary environments, but similar to some hydrothermal vent structures (Table S1, Figure S2). Thus, some clades of Hydrothermarchaeota may be well adapted to life in the warm crustal biosphere, and detailing the potential metabolisms of these organisms should advance our understanding as to how life survives in this energy-limited environment.

This study combined the analysis of Hydrothermarchaeota metagenome-assembled genomes (MAGs [15]) with several newly generated SAGs from the same JdFR crustal fluids to evaluate Hydrothermarchaeota’s evolutionary relationship to other archaeal groups and assess the functional potential of the lineage. Our data reveal an early-branching archaeal candidate phylum arising between the Euryarchaeota superphyla and the superphylum containing Micrarchaeota, Altiarchaeota, UAP2, and Nanoarchaeota (DPANN (Diapherotrites, Parvarchaeota, Aenigmarchaeota, Nanoarchaeota, and Nanohaloarchaeota) [25, 26]). This basal evolutionary position is supported by the coding potential for early-evolved enzymes for anaerobic sulfate and nitrate reduction.

## Materials and methods

### Observatory description and fluid sampling

During the IODP Expedition 327 in 2010, borehole observatories were placed in subseafloor basement at IODP Holes U1362A and U1362B (Table S2, Figure S1). These observatories feature epoxy-coated steel observatory casing to minimize corrosion and mitigate impact on in situ processes, Teflon-lined “umbilical” tubes for pristine fluid collection from isolated subsurface intervals, and a 4-inch diameter “free flow” ball valve at the wellhead for additional fluid sampling [27–29]. Each borehole penetrates approximately 235 m of sediment. Hole U1362B has an umbilical for fluid collection 30 m below the sediment-basement interface (meters sub-basement (msb)), while Hole U1362A has fluid collection horizons at approximately 30 and 190 msb.

In July 2011, fluid samples were collected using equipment on the remotely operated vehicle (ROV) *Jason II* from the Research Vessel *Atlantis* (Table S2). Fluids for metagenomic analyses were sampled from the umbilicals that accessed 190 and 30 msb at Holes U1362A and U1362B, respectively, as described previously [15]. At the seafloor, a mobile pumping system filtered approximately 124 and 70 l of crustal fluid from boreholes U1362A and U1362B using Steripak-GP20 (Millipore, Billerica, MA, USA) polyethersulfone filter cartridges containing 0.22 μm pore-sized membranes [15]. Before filtering, at least three times the volume of the umbilical line was flushed through the system to remove any stagnant fluids. Fluids for single-cell genomic analyses were sampled from the 190 msb Hole U1362A using the same mobile pumping system [23] and from the open ball valve on the wellhead at Hole U1362B [16], after a long-period of free flow to flush out the borehole dead volume, using a syringe cleaned with bleach and dilute trace-metal-grade acid. Temperatures as high as 62 °C were recorded with the ROV thermistor inside the ball valve opening. Immediately upon recovery, fluid was fixed with glycerol-Tris-EDTA (glyTE) buffer and frozen at −80 °C in cryovials for single-cell sorting [30].

### Single-cell sorting, genome sequencing, and assembly

The generation, identification, sequencing, and de novo assembly of SAGs was performed at the Bigelow Laboratory for Ocean Sciences Single Cell Genomics Center (scgc.bigelow.org). The cryopreserved samples were thawed, pre-screened through a 40 μm mesh size cell strainer (Becton Dickinson) and incubated with 5 μM (final concentration) SYTO-9 DNA stain (Thermo Fisher Scientific) for 10–60 min. Fluorescence-activated cell sorting, cell lysis, multiple displacement amplification, sequencing (using Illumina technology), de novo genome assemblies, and quality control were performed using the workflow benchmarked in ref. [30]. Contigs >2 kbp in length were uploaded to the Joint Genome Institute (JGI) Integrated Microbial Genomes & Microbiomes (IMG/M) comparative data analysis system (Table S3 [31]) for gene prediction and annotation using the genome annotation pipeline [32].

### Metagenome sequencing, assembly, and annotation

Metagenome sequencing, assembly, binning, and annotation has been reported previously [15]. Briefly, quality-filtered raw sequence reads from the crustal fluids of Hole U1362A (IMG/M ID 330002481) and Hole U1362B (IMG/M ID 3300002532) were assembled using SOAPdenovo version 1.05 with default settings, binned using CONCOCT [33] and curated within the Anvi’o package, version 1.1.0 [34]. In total, 98 MAGs were produced, of which 3 were identified as Hydrothermarchaeota. Hydrothermarchaeota MAGs JdFR-16, JdFR-17, and JdFR-18 were uploaded to IMG/M for gene prediction and annotation using the genome annotation pipeline [32]. Completeness and contamination estimates for SAGs and MAGs were made by comparing annotated protein sequences against the Euryarchaeota marker list within CheckM [35]. Average Nucleotide Identity (ANI) comparisons were calculated using IMG/M pairwise ANI tool [31].

### Phylogenetic and phylogenomic analyses

Phylogenetic trees of the 16S rRNA, nitrate reductase, and ribulose-1,5-bisphosphate carboxylase/oxygenase (RuBisCO) genes were constructed using raxMLHPC (version 8.2.8, [36], see supplemental information). Briefly, 16S rRNA genes were aligned using the SILVA Incremental Aligner (SINA) online tool [37] and masked out with the lane1349 mask [38]. Nitrate reductase and RuBisCO genes were aligned using MUSCLE (version 3.8.31 [39]) and trimmed and masked using trimAl (version 1.2rev59 [40]). All alignments were manually inspected.

A phylogenomic tree based on 43 single-copy marker genes was created from all publicly available genomes from the archaeal domain from IMG/M, National Center for Biotechnology Information (NCBI), and other repositories of data (Tables S4, S5). MAGs and SAGs with CheckM-generated completeness, contamination, and strain heterogeneity information (version 1.0.11 [35]) were used as input to dRep (version 2.0.5 [41]) to produce a dereplicated set of genomes for phylogenomic analysis. Most default dRep parameters were used, but the required completeness was reduced to 50% to account for genes that are systematically absent from single-copy marker gene sets in archaeal groups of critical importance to the phylogeny (e.g., Hadesarchaeaota). From the dereplicated genomes (*n* = 1198) and the three most complete Hydrothermarchaeota genomes (JdFR-17, JdFR-18. and SAG AC-708-L17), the 43 single-copy marker gene alignment produced by CheckM was used as input to FastTree [42] with the WAG amino acid substitution model. Phylogenetic lineages were identified and collapsed in ARB (version 6.0.4 [43]) with the guided assistance of taxonomic information generated using GTDB-Tk (version 0.0.7) using the classify workflow with database release 83 [26].

### Amplification of *mcrA* gene

Amplification of the *mcrA* gene on a sorted, whole genome-amplified Hydrothermarchaeota cell was attempted using primers qmcrA [44] and ML 5 ([45], see supplemental information).

### Thermodynamic calculations of Gibbs free energy

The potential energy yields for sulfate reduction coupled to various electron donors were calculated according to the Gibbs energy of reaction (see supplemental information).

## Results and discussion

### Comparative genomics of Hydrothermarchaeota MAGs and SAGs

Hydrothermarchaeota constituted 42% (*n* = 28/66) and 24% (*n* = 23/94) of the identified SAGs from the Holes U1362A and U1362B, respectively. Five SAGs were chosen for genome sequencing: SAGs AC-708-L17 and AC-708-N22 from Hole U1362A; and AC-334-K11, AC-335-G21, and AC-335-L21 from Hole U1362B (Table 1). These SAGs range in size and estimated completeness from 1.26 Mbp and 70% complete (AC-708-L17) to 0.47 Mbp and 23% complete (AC-708-N22; Table 1). Based on these SAGs, a complete Hydrothermarchaeota genome is estimated to approximate 1.8 Mbp, comparable to other subsurface Archaea [46, 47]. The three Hydrothermarchaeota MAGs previously constructed from Holes U1362A (MAGs JdFR-17 and JdFR-18) and U1362B (MAG JdFR-16 [15]) range from 1.35 Mbp and 31% complete to 2.18 Mbp and 97% complete (Table 1). Contamination estimates (sequence redundancy) within the MAGs range from 7 to 25%, although strain heterogeneity estimates (72–80% [35]) suggest that the redundancy may reflect the binning of closely related Hydrothermarchaeota strains. Genomic guanine–cytosine (GC) content is approximately 50% for all genomes except MAG JdFR-18, which is 39%. The five Hydrothermarchaeota SAGs have similar 16S rRNA genes (>99%, Table S6). Of the two MAGs that contained 16S rRNA genes, JdFR-17 was >99% similar to the five Hydrothermarchaeota SAGs, while the JdFR-18 16S rRNA gene was only 88–89% similar to the Hydrothermarchaeota SAGs and MAG JdFR-17 (Table S6), potentially representing a second Hydrothermarchaeota family.

**Table 1:** General characteristics of *Candidatus* Hydrothermarchaeota SAGs and MAGs

	Single-amplified genomes (SAGs)					Metagenome-assembled genomes (MAGs)		
Genome ID	AC-334-K11	AC-708-L17	AC-335-L21	AC-335-G21	AC-708-N22	JdFR-16	JdFR-17	JdFR-18
Isolation source	U1362B	U1362A	U1362B	U1362B	U1362A	U1362B	U1362A	U1362A
Assembly size (mbp)	0.92	1.26	0.73	0.63	0.47	1.35	2.18	2.06
GC content (%)	50.9	50.7	51.0	50.5	50.3	49.7	49.6	39.1
No. of contigs	33	39	23	24	23	241	334	22
Max contig length (kbp)	179	191	125	129	70.6	45.2	39.6	364
N50 (bases)	59,108	70,385	64,756	68,317	34,461	6267	7687	149,032
Predicted gene count	1129	1499	877	763	543	1715	2771	2320
Estimated genome completeness (%)	43.2	69.5	48.8	39.2	22.9	31.1	53.9	96.8
Estimated contamination (%)	0.0	1.6	0.8	0.0	0.0	7.3	25.2	2.4
Estimated strain heterogeneity	--	--	--	--	--	80.8	73.2	72.2
MISAG/MIMAG quality^a^	Low	Medium	Low	Low	Low	Low	Low	High
16S rRNA gene presence	Yes	Yes	Yes	Yes	Yes	No	Yes	Yes
16S rRNA gene length (bp)	526	1489	1489	517	1,447	NA	1086	1476

^a^Draft genome quality determined as suggested by Bowers et al. [83]

**Fig. 1:**
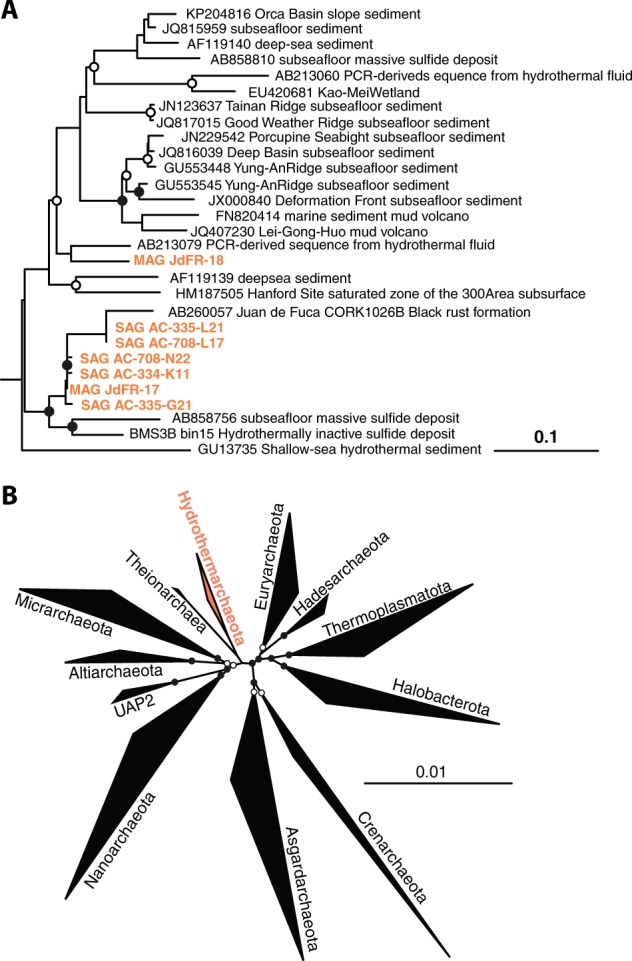
Phylogenetic associations of the Juan du Fuca *Candidatus* Hydrothermarchaeota. Black (100%) and white (99–80%) circles indicate nodes with high local support values. **a** Phylogenetic associations relative to other *Ca*. Hydrothermarchaeota. 16S rRNA genes sequences from this study are in bold, sequences from other studies are indicated with their accession numbers (Table S8). **b** Phylogenomic associations of *Ca*. Hydrothermarchaeota genomes among archaeal genomes publicly available in Integrated Microbial Genomes (IMG), National Center for Biotechnology Information (NCBI), and other repositories, using classifications suggested by the Genome Taxonomy Database [26] (Table S4). Tree represents the concatenation of 43 single copy marker proteins (Table S5)

Phylogenetic analyses revealed that the JdFR Hydrothermarchaeota 16S rRNA gene sequences grouped most closely with environmental sequences from other crustal environments (Fig. 1a). The majority of sequences clustered with a sequence from black rust that formed on the exterior of a leaking subseafloor observatory at nearby Hole 1026B, where the black rust was still exposed to hydrothermal fluids leaking from the observatory [48]. MAG JdFR-18 branched separately with a sequence identified within crustal fluids collected from the hydrothermal vent of the Southern Mariana Trough [49].

Genome-wide ANI values corroborate the 16S rRNA gene phylogeny (Table S7). With the exception of SAG AC-708-N22, the SAGs are very similar (mean 98.6 ± 1.4% s.d.; *n* = 4). SAG AC-708-N22 is most similar to MAG JdFR-17, sharing an ANI value of 98.2%. Consistent with the 16S rRNA gene phylogeny and GC content, MAG JdFR-18 is equally dissimilar to all other genomes (mean 68 ± 0.8% s.d.; *n* = 8). These genomic relationships highlight the connectivity between the two different borehole locations and depth horizons (Table S2) and the diversity within the Hydrothermarchaeota community.

### Evolutionary placement of the Hydrothermarchaeota

This study sheds light on the taxonomic placement of Hydrothermarchaeota within the archaeal tree of life. Phylogenies based on 16S rRNA genes as well as concatenated alignments of 43 single-copy phylogenetic marker genes place the Hydrothermarchaeota lineage branches toward the root of the DPANN superphylum (Fig. 1b). Currently, there are several predictions for the root of the archaeal tree, including placement within the Euryarchaeota [4, 50] or between DPANN and the remaining archaeal lineages [5]. Regardless, the placement of Hydrothermarchaeota [26] between Euryarchaeota and DPANN lineages suggest that Hydrothermarchaeota represents an early-branched lineage that should be considered in future evolutionary models. Likewise, additional exploration of the JdFR crustal ecosystem is important for interpreting the metabolic potential of this early-branched lineage. Similar to other proposed early-life analogs, the crustal aquifer presents a hot, anoxic environment protected from sunlight and oxygen [51], but is drastically understudied relative to hydrothermal vent systems.

### Terminal electron acceptors for Hydrothermarchaeota

**Fig. 2:**
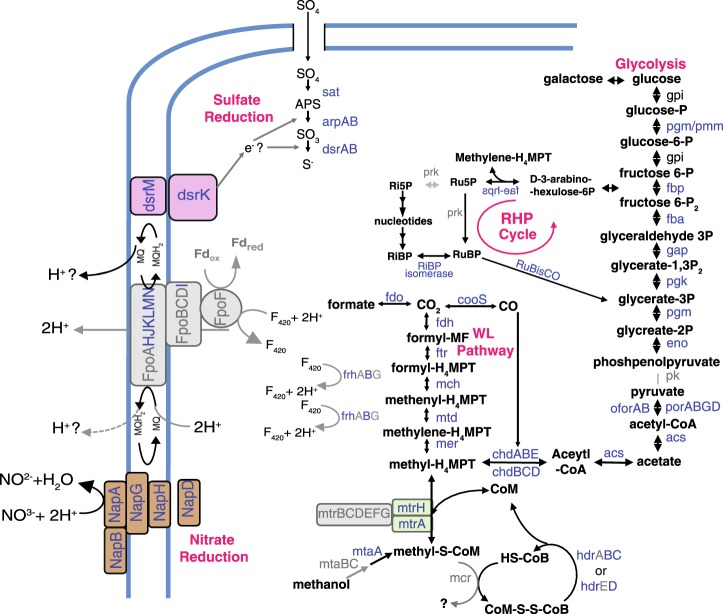
Metabolism interpretation of *Candidatus* Hydrothermarchaeota single-amplified genomes (SAGs) and metagenome-assembled genomes (MAGs) from Juan de Fuca Ridge flank subsurface crustal aquifer, based on the genes present within all genomes collectively. Black labels represent metabolites, blue labels represent genes or gene subunits that are present within at least one of the genomes (for individual genomes see Figure S4), gray labels represent genes or subunits not found in the genomes studied. Two black arrows aligned in the same direction represent a pathway requiring multiple genes, all of which were found in at least one genome. Pathway abbreviations: WL Wood–Ljungdahl, RHP reductive hexulose-phosphate. Gene name abbreviations: cdhABCDE CO dehydrogenase/acetyl-CoA synthase (subunits alpha, A; epsilon, B; beta, C; delta, D; gamma, E), cooC CO dehydrogenase maturation factor, cooS carbon monoxide dehydrogenase catalytic subunit, fwdABCDEFG formylmethanofuran dehydrogenase (subunits A–G), ftr formylmethanofuran-tetrahydromethanopterin formyltransferase, mch methenyltetrahydromethanopterin cyclohydrolase, mtd methylenetetrahydromethanopterin dehydrogenase, mer methylenetetrahydromethanopterin reductase, mtrA tetrahydromethanopterin *S*-methyltransferase (subunit A), hdrBCD CoB–CoM heterodisulfide reductase (subunits B–D), fdo formate dehydrogenase, Fqo ferredoxin:NADP^+^ oxidoreductase, frhABG coenzyme F420-reducing hydrogenase (subunits ABG), pgm/pmm phosphomannomutase/phosphoglucomutase, gpi glucose-6-phosphate isomerase, fba fructose-bisphosphate aldolase, fbp D-fructose 1,6-bisphosphatase, gap glyceraldehyde 3-phosphate dehydrogenase, pgk phosphoglycerate kinase, pgm phosphoglycerate mutase, eno enolase, pk pyruvate kinase, porABGD pyruvate ferredoxin oxidoreductase (subunits A–D), acs acetyl-coenzyme A synthetase, apr dissimilatory adenylylsulfate reductase (subunits A, B), dsrAB sulfite reductase alpha (subunits, A, B), sat sulfate adenylyltransferase, NapADGH nitrate reductase (subunits ADGH). Biomolecule abbreviations: SO_4_ sulfate, APS adenosine-5’-phosphate, SO_3_ sulfite, S sulfide, NO_3_^−^ nitrate, NO_2_^−^ nitrite, MQ menaquinone, F420 coenzyme F420, MF methanofuran, MPT methanopterin, CoA/CoB/CoM coenzyme A/B/M, P phosphate

By leveraging metagenomics with single-cell genomics, we were able to validate the binning of the previously constructed MAGs by confirming the presence of important genes within the partial SAGs. Here, the functional attributes of each genome are evaluated individually (Figure S4) in order to provide a collective summary of the lineage’s metabolic potential (Fig. 2).

The Hydrothermarchaeota genomes contain genes for the use of several different terminal electron acceptors including sulfate, nitrate, and potentially metal oxides (Figure S4), suggesting versatility in the choice of oxidant. Phylogenetic analyses of key sulfate and nitrate reductase subunits suggest that these genes represent some of the earliest evolved forms of these enzymes found to date, further supporting Hydrothermarchaea as a deeply branching lineage (Figure S5 [52]).

The capacity for sulfate reduction is evident in the JdFR Hydrothermarchaeota (Fig. 2, Table S9). Most of the genomes had at least one *sulP* permease, suggesting that sulfate can readily enter Hydrothermarchaeota cells, while MAG JdFR-18 also included coding regions for sulfate adenylyltransferase (sat), the enzyme responsible for reducing sulfate to adenosine-5′-phosphosulfate (APS), and APS reductase for reducing APS to sulfite. The identification of genes for dissimilatory sulfite reductase (*dsrAB*) in SAG AC-708-L17 and the three MAGs suggests that sulfite reduction to sulfide is also likely. The *dsrA* genes found in SAG AC-335-L21 and the three MAGs are most closely related to *dsrA* from *Moorella* species, “*Candidatus* Rokubacteria”, and “*Candidatus* Aigarchaeota”, and appear to be an early-evolved form of the sulfate reductase gene, as suggested previously [52]. The *dsrA* genes of Hydrothermarchaeota are not monophylogenetic: additional *drsA* genes present in MAG JdFR-18, and SAGs AC-334-L17 and AC-334-K11 are similar to genes from bacteria and may be obtained via horizontal gene transfer [52]. The electrons for sulfate reduction are possibly passed from the menaquinone loop using membrane-bound *dsrMK*, and then to a soluble, unidentified electron carrier protein. For example, the protein DsrC has been suggested to be an electron carrier in past studies of *Archaeoglobus* [53]. The possible roles of other sulfur species as electron donors or acceptors for Hydrothermarchaeota are limited, and no evidence of thiosulfate reductase was found in any of the Hydrothermarchaeota genomes.

There is ample evidence for microbial sulfate reduction within the JdFR crustal fluids. Fluids are replete with sulfate (~18 mM [19, 20]), demonstrate measurable sulfate reduction, and contain dissimilatory *dsrAB* genes [54]. *dsrAB* genes were also observed in JdFR rocks, along with pyrite sulfur stable isotope values that indicate microbial sulfate reduction [55]. We hypothesize that Hydrothermarchaeota contribute to the sulfate reduction potential in this ecosystem, along with the Deltaproteobacteria, Firmicutes, and *Archaeoglobus* microbial community members previously identified in this ecosystem [15, 54].

Hydrothermarchaeota may have metabolic flexibility in terminal electron acceptors for respiration, as evidenced by the presence of genes for nitrate reduction. Genomes contain subunits for two different types of nitrate reductase genes: *nap*, the periplasmic dissimilatory nitrate reductase genes, and *nar*, the cytoplasmic membrane-bound nitrate reductase (Fig. 2, S4, Table S10). Phylogenetic analysis of *napA* genes suggests that the Hydrothermarchaeota *napA* gene represents an early-evolved form, supporting other evidence for Hydrothermarchaeota’s basal placement in the archaeal tree of life (Figure S5). However, the possibility for nitrate reduction in this ecosystem is unclear. Nitrate is rapidly exhausted after entering the ocean crust [18] and measured concentrations within the JdFR fluids over multiple years have been below detection or at nanomolar concentrations [19, 20]. Plausibly, trace amounts of nitrate could be intensely cycled, as has been observed in continental subsurface systems with cryptic N cycling [56] and suggested from other JdFR metagenomic interpretations [15]. Thus, the possibility of nitrate reduction in this crustal ecosystem warrants further attention.

Most genomes also possess various subunits for cytoplasmic membrane-bound nitrate reductase (subunits *narGHIJ*, Table S10). The carboxydrotroph *A. fulgidus* also has this nitrate reductase, but has not demonstrated nitrate reduction in the laboratory [53]. Interestingly, transcripts of *A. fulgidus* show an upregulation of *narA* when reducing sulfate, indicating that this nitrate reductase complex might be accepting electrons from ferredoxin to reduce menaquinone [53]. However, additional laboratory studies are necessary to decipher the true potential of nitrate reductase in *A. fulgidus* and JdFR Hydrothermarchaeota.

Hydrothermarchaeota genomes include genes for many different *c*-type cytochromes, which are likely involved in terminal electron transferring processes (Table S11). Within the Archaea, microorganisms known to contain *c*-type cytochromes are restricted to the orders Archaeoglobales, Methanosarcinales, Halobacteriales, and Thermoplasmatales [57], where they serve as electron transfer proteins. Given the metabolisms of neutrophilic anaerobes within these orders, we hypothesize that the *c*-type cytochromes in Hydrothermarchaeota are either (1) generating a proton gradient through the use of an electron transport system, as observed in methanogens and methanotrophs of the Methanosarcinales order [57], or (2) reducing extracellular ferric oxide species, as observed in *Ferroglobus placidus* and *Geoglobus ahangari* [57], iron reducing archaea of other marine hydrothermal systems.

### Carbon cycling by Hydrothermarchaeota

All Hydrothermarchaeota genomes have genes for carbon monoxide (CO) cycling and the Wood–Ljungdahl pathway (Fig. 2, Tables S12,13), suggesting that these organisms are carboxydotrophs capable of using CO in dissimilative and assimilative pathways [58]. Most genomes contained the mono-functional CO dehydrogenase catalytic subunit (*cooS*) and/or the CO dehydrogenase maturation factor genes (*cooC*), which may be used together to oxidize CO to CO_2_ for dissimilative processes [59]. The Hydrothermarchaeota genomes lack evidence for CO-induced hydrogenase subunits (*cooH* and *cooL*) and associated electron carrier (*cooF*) used by bacterial hydrogenogenic carboxydotrophs *Rhodospirillum rubrum* and *Carboxydothermus hydrogenoformans* [60, 61]. Instead, Hydrothermarchaeota probably couple CO oxidation to sulfate reduction similar to *Archaeoglobus fulgidus* [62].

Gene subunits for the ferredoxin:NADP^+^ oxidoreductase (*fqo*) complex may represent a potential electron shunt into the membrane-bound respiratory chain, linking CO oxidation to an external electron acceptor (Table S14 [53, 63]). Genes for the biosynthesis of menaquinone suggests that menaquinone redox reactions could continue to transfer elections along the respiratory chain to an ultimate acceptor, while translocating protons across the membrane (Table S14).

Hydrothermarchaeota SAGs and MAGs also contain one to two bifunctional CO dehydrogenase/acetyl-CoA synthase (CODH/ACS) complexes (*cdhABC* and *cdhCDE*; Fig. 2, S12), which couple the reversible reduction of CO_2_ to CO oxidation to form acetate (acetogenesis), a key step of the Wood–Ljungdahl pathway [64]. The CODH/ACS complex is hypothesized to be an early-evolved complex [58], and thus its presence in this early-branching lineage is consistent with its proposed ancestry. The presence of these complexes and other Wood–Ljungdahl enzymes suggest that the Hydrothermarchaeota can also assimilate carbon monoxide to biomass, again similar to *A. fulgidus*. Several of the SAGs and the three MAGs also contain formate dehydrogenase (Fig. 2, Table S13), suggesting that formate production during CO oxidation or the use of formate as an electron donor is possible, as observed with cultures of *A. fulgidus* [62, 65].

Hydrothermarchaeota do not appear to be involved in methane cycling, although they possess some genes known to be involved in methyl cycling. For example, the genomes contain genes that encode for the methanogenic tetrahydromethanopterin *S*-methyltransferase subunit (*mtrH*, Table S13), and MAG JdFR-18 also encodes genes for methyl transferases of methylamide compounds (Table S15). These enzymes are all involved with the methylation of methyl-coenzyme M (methyl-SCoM); during methanogenesis, methanogens reduce methyl-SCoM with the enzyme methyl-coenzyme M reductase (MCR). No subunits of the *mcr* gene were found within the Hydrothermarchaeota genomes, suggesting that these organisms cannot produce methane. To verify that the absence of the *mcr* subunits did not result from a lack of recovery, a PCR reaction targeting the *mcrA* gene was performed on amplified SAG DNA from a sorted cell that was identified as Hydrothermarchaeota but not genome sequenced. No PCR product was observed (data not shown). A negative result supports the absence of *mcrA* in these Hydrothermarchaeota genomes, but cannot rule out possible biases related to the amplification reactions. Nevertheless, the presence of *mtrH* and lack of *mcrA* has been recognized in Theionarchaea (SAG DG-70, [66]) and Euryarchaeota genomes (*A. fulgidus*, IMG/M IDs 2588253768/ 638154502; *Achaeoglobus sulfacticallidus*, IMG/M ID 2522125074, and a partial MSBL1 genome [67]). This suggests that the methyl-SCoM metabolite may have an alternate fate in these organisms. Given that the last common ancestor has been hypothesized to be a methanogen [3, 68], the placement of Hydrothermarchaeota outside the Euryarchaeota (many of which are methanogens) may further advance our understanding of the distribution and evolution of this ancient and fundamental metabolism.

While no known CO-induced hydrogenases were identified, genes for Ni-Fe hydrogenases (large and small subunits) were found in SAG AC-344-K11 and MAGs JdFR-16 and JdFR-17 (Fig. 2, Table S16). These hydrogenases may serve as a sink for the electrons produced during CO oxidation. However, it is also possible that the Ni-Fe hydrogenases may oxidize hydrogen for the hydrogenotrophic reduction of sulfate, as previously observed as an alternative to carbon monoxide oxidation in a culture of *A. fulgidus* [69]. In addition to Ni-Fe hydrogenases, most JdFR Hydrothermarchaeota contained genes encoding for the F420-reducing hydrogenase beta subunit, a hydrogenase required for the Wood–Ljungdahl pathway, as well as genes for hydrogenase biosynthesis proteins (hypABCDEF) and hydrogenase maturation proteins.

**Fig. 3:**
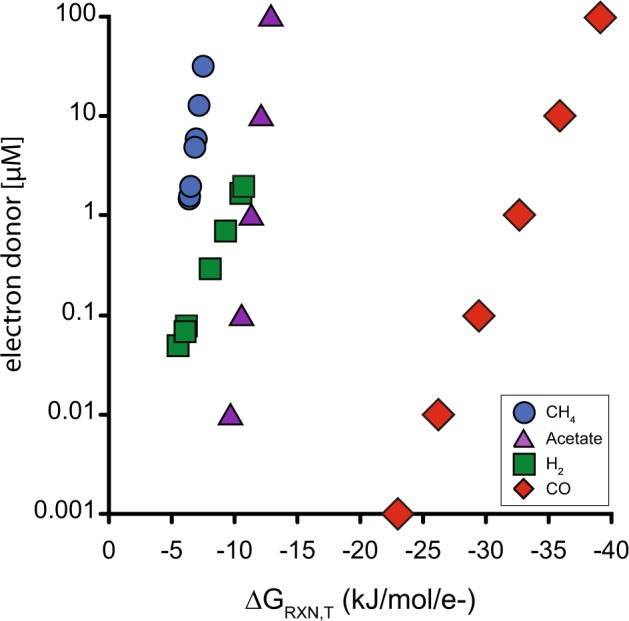
Free energy yield (kJ mol^–1^ e^–1^) for sulfate reduction coupled to acetate, hydrogen, methane, or carbon monoxide (CO) oxidation (Table S19) at various electron donor concentrations, based on the in situ conditions of Juan de Fuca Ridge fluids (Table S18)

Given the evidence for CO oxidation and sulfate reduction, Gibbs free energy yields were estimated for sulfate reduction with CO oxidation and compared to various other electron donors (equation S1, Tables S17-S19), following approaches described previously [70–72]. When possible, in situ concentrations were used for the calculations (Table S18 [19, 71]). To our knowledge, concentrations of CO and acetate have not been measured within JdFR crustal fluids in this ecosystem; thus, calculations were based on a range of concentrations for these analytes from (10 nM to 100 µM, based on reported in situ CO concentrations in other marine and fluid-rock reaction environments ([73, 74] Table S18). Of the reactions tested, CO oxidation coupled with sulfate reduction yielded the most exergonic conditions when normalized per electron transferred (Fig. 3, Table S19). This supports the interpretation of dissimilatory carboxydotrophy metabolism in Hydrothermarchaeota, which, as an autotroph may feed the microbial food web and drive the carbon cycling in this warm crustal biosphere.

### Metabolic pathways for essential metabolites and biomolecules

The JdFR Hydrothermarchaeota contain pathways for sugar, amino acids, nucleic acids, and lipid metabolism. Collectively, the genomes have many of the genes required for the gluconeogenesis/glycolysis pathway, missing only pyruvate kinase (Table S20). Instead of being converted to pyruvate, we hypothesize that phosphoenolpyruvate is likely converted to oxaloacetate by phosphoenolpyruvate carboxylase. The presence of fumarate hydratase, succinate dehydrogenase, succinyl-CoA synthetase, 2-oxogluterate ferredoxin reductase, and isocitrate dehydrogenase suggests the presence of an incomplete tricarboxylic acid (TCA) cycle (Table S21), which is similar to other anaerobic archaeal groups such as methanogens [75]. In anaerobic organisms, TCA-related genes provide for the potential synthesis of several important biosynthetic intermediates such as fumarate, succinate, succinyl-CoA, and 2-oxoglutate. These intermediates can then serve as the building blocks for amino acid, pyrimidine, and purine metabolisms (Table S22). The Hydrothermarchaeota possess many genes for synthesis of isoprenoid-based lipids using the mevalonate pathway, including hydroxymethylglutaryl-CoA synthase, isopentenyl phosphate kinase, isopentenyl-diphosphate delta-isomerase and mevalonate kinase (Table S23). Transporters for trace elements (Co, Ni, Mo, W) and the vitamin biotin were identified, along with transporters for branched amino acids (Table S24). These could then serve as a potential source of nitrogen and organic carbon for the cell.

Some JdFR Hydrothermarchaeota genomes contain RuBisCO genes (Fig. 2), which were aligned against other RuBisCO genes to understand their potential metabolic function. These Hydrothermarchaeota RuBisCO genes group phylogenetically with form III-a RuBisCOs (Figure S6), which are known to fix CO_2_ for the synthesis of metabolites (including nucleic acids and sugars) using the reductive hexulose-phosphate (RHP) cycle [76]. Hydrothermarchaeota include many of the genes necessary for the RHP cycle, including a gene for a fused hexulose-6-phosphate/formaldehyde activating enzyme (Table S25). When these two enzymes act in concert, they produce methylene-H_4_MPT from 3-arbino-hexulose-6-phosphate, an important metabolite for the Wood–Ljungdahl pathway. However, phosphoribulokinase, an important RHP enzyme, has yet to be identified within the JdFR Hydrothermarchaeota genomes, and thus the potential for the RHP cycle cannot be confirmed.

### Motility as an adaptive strategy of Hydrothermarchaeota

**Fig. 4:**
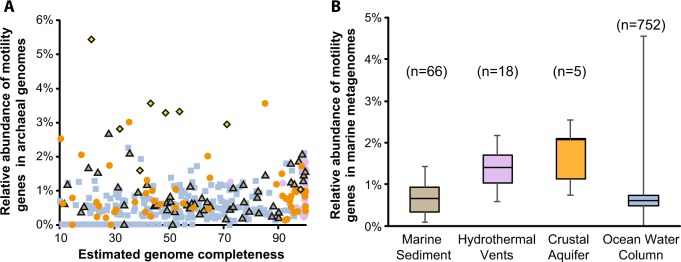
The relative abundance of motility genes in publicly available marine-related genomes and metagenomes as defined by COG (Clusters of Orthologous Groups) annotations. **a** Relative abundance of motility genes in single-amplified genomes and metagenome-assembled genomes collected from various marine environments relative to their genome completeness (considering genomes that are at least 10% complete, Table S28): sediments (brown triangle), hydrothermal vents (purple circle), crustal aquifers (orange circles), and ocean water column samples (blue squares). *Candidatus* Hydrothermarchaeota genomes are highlighted as yellow diamonds. **b** The relative abundance of motility genes in metagenomes collected from various marine environments (Table S29). The box and whiskers represent the range of relative abundance as defined by quartiles

JdFR Hydrothermarchaeota partial genomes contain more chemotaxis and motility-related genes than many of the archaeal SAGs publicly available in IMG/M (Table S26-27). Specifically, when compared to archaeal genomes of marine environments, and accounting for relative completeness, JdFR genomes generally have more motility genes than genomes from sedimentary environments (Fig. 4a). A similar trend can be observed when comparing community-wide metagenomic samples (Fig. 4b). The relative abundance of motility genes within metagenomes from crustal environments (Juan de Fuca and the Mid-Atlantic Ridge [77]) are greater than sediment environments and comparable to ocean water column samples.

Motility genes found in the Hydrothermarchaeota genomes include those for the archaellum and chemotaxis. It is unclear if Hydrothermarchaeota use the archaellum solely for motility or to attach to surfaces, leading to biofilm formation. The processes for attachment are diverse within Archaea [78], and thus the presence of archaellum-related genes do not necessarily indicate or exclude potential biofilm production. In either case, archaellum rotation would likely be driven by adenosine triphosphate hydrolysis [79]. This poses a problem for microorganisms living in subsurface low-energy environments, which are thought to be surviving just slightly above their minimum energy requirements [72, 80]. Subsurface cells are expected to focus on maintenance over growth, suggesting that motility would be impractical [80]. However, the possibility of motility in sediment is suggested from metatranscriptomes analyzed from sediments of the Peru Margin [81]. This prior work suggested that motility decreases with decreasing porosity, a trend that supports the comparisons of Fig. 4. Given that the crustal biosphere is relatively porous compared to a sedimentary environment, the advantages of motility or biofilm production in this fluid environment can be appreciated, even though the energy requirements remain a paradox. Assuming that nutrients and concentrations of electron acceptors exist as patches that disperse with time (i.e., decaying cells, marine snow particles), the energetic gain of motility would depend on the size and concentration of the chemoattractant, degree of fluid mixing, the distance between the cell and nutrient packet, and the speed at which a cell could travel to the nutrient packet [82]. Alternatively, surface attachment and biofilm production can provide protection and foster metabolic interdependencies [78]. Either way, the abundance of genes related to motility and chemotaxis in these Hydrothermarchaeota genomes, and crustal metagenomes in general, suggest that organisms of the deep crustal biosphere have adopted a strategy for balancing these energetic gains and costs.

## Conclusions

Single-cell and metagenome-assembled genomes from the uncultivated Hydrothermarchaeota lineage were derived from warm, anoxic subsurface crustal fluids collected from the Juan de Fuca Ridge flank. Comparative genomic analysis provided the first evolutionary and metabolic characterization of this lineage. These genomic datasets revealed Hydrothermarchaeota to be an early-branching archaeal phylum, arising near the central branch points of the DPANN and Euryarchaeota groups. The genomes harbor evidence for many early-evolved metabolisms including ancient forms of sulfate and nitrate reductases. These observations underscore the significance of the hot, deep marine crustal biosphere as an important habitat for understanding the evolution of early life. Hydrothermarchaeota appear to be carboxydotrophs, highlighting this often-overlooked metabolic pathway as playing an important role in subsurface fluid-rock reaction environments, as suggested recently [73]. The presence of chemotactic and motility genes suggests that Hydrothermarchaeota may be capable of seeking favorable redox conditions or other nutrients. Despite a small average genome size, the versatility afforded by the inferred metabolic and phenotypic characteristics of the Hydrothermarchaeota may represent important survival strategies for life in the warm crustal biosphere.

## Supplementary information

Supplemental Material

Supplemental Excel File

## References

[1] Adam PS, Borrel G, Brochier-Armanet C, Gribaldo S (2017). The growing tree of Archaea: new perspectives on their diversity, evolution and ecology. ISME J.

[2] Spang A, Caceres EF, Ettema TJG (2017). Genomic exploration of the diversity, ecology, and evolution of the archaeal domain of life. Science.

[3] Borrel G, Adam PS, Gribaldo S (2016). Methanogenesis and the Wood–Ljungdahl pathway : an ancient, versatile, and fragile association. Genome Biol Evol.

[4] Raymann K, Brochier-armanet C, Gribaldo S (2015). The two-domain tree of life is linked to a new root for the Archaea. Proc Natl Acad Sci USA.

[5] Williams Tom A., Szöllősi Gergely J., Spang Anja, Foster Peter G., Heaps Sarah E., Boussau Bastien, Ettema Thijs J. G., Embley T. Martin (2017). Integrative modeling of gene and genome evolution roots the archaeal tree of life. Proceedings of the National Academy of Sciences.

[6] Hug LA, Baker BJ, Anantharaman K, Brown CT, Probst AJ, Castelle CJ (2016). A new view of the tree of life. Nat Microbiol.

[7] Pace NR (2009). Mapping the tree of life: progress and prospects. Microbiol Mol Biol Rev.

[8] Spang A, Saw JH, Jørgensen SL, Zaremba-Niedzwiedzka K, Martijn J, Lind AE (2015). Complex archaea that bridge the gap between prokaryotes and eukaryotes. Nature.

[9] Zaremba-Niedzwiedzka K, Caceres EF, Saw JH, Bäckström D, Juzokaite L, Anantharaman K (2017). Asgard archaea illuminate the origin of eukaryotic cellular complexity. Nature.

[10] Evans PN, Parks DH, Chadwick GL, Robbins SJ, Orphan VJ, Golding SD (2015). Methane metabolism in the archaeal phylum Bathyarchaeota revealed by genome-centric metagenomics. Science.

[11] Jay ZJ, Beam JP, Dlakić M, Rusch DB, Kozubal MA, Inskeep WP (2018). ﻿Marsarchaeota are an aerobic archaeal lineage abundant in geothermal iron oxide microbial mats. Nat Microbiol.

[12] Baker BJ, Saw JH, Lind AE, Lazar CS, Hinrichs K, Teske AP (2016). Genomic inference of the metabolism of cosmopolitan subsurface Archaea, Hadesarchaea. Nat Microbiol.

[13] Chuvochina Maria, Rinke Christian, Parks Donovan H., Rappé Michael S., Tyson Gene W., Yilmaz Pelin, Whitman William B., Hugenholtz Philip (2019). The importance of designating type material for uncultured taxa. Systematic and Applied Microbiology.

[14] Vetriani C, Jannasch HW, MacGregor BJ, Stahl DA, Reysenbach AL (1999). Population structure and phylogenetic characterization of marine Benthic archaea in deep-sea sediments. Appl Environ Microbiol.

[15] Jungbluth SP, Amend JP, Rappé MS (2017). Data Descriptor: metagenome sequencing and 98 microbial genomes from Juan de Fuca Ridge flank subsurface fluids. Sci Data.

[16] Fisher AT, Tsuji T, Petronotis K, Wheat CG, Becker K, Clark JF (2012). IODP Expedition 327 and Atlantis Expedition AT 18-07: observatories and experiments on the Eastern Flank of the Juan de Fuca Ridge. Sci Drill.

[17] Elderfield H, Wheat CG, Mottl MJ, Monnin C, Spiro B (1999). Fluid and geochemical transport through oceanic crust: a transect across the eastern flack of the Juan de Fuca Ridge. Earth Planet Sci Lett.

[18] Wheat CG, Hulme SM, Fisher AT, Orcutt BN, Becker K (2013). Seawater recharge into oceanic crust: IODP Exp 327 Site U1363 Grizzly Bare outcrop. Geochem Geophys.

[19] Wheat CG, Jannasch HW, Fisher AT, Becker K, SharkeyJ, Hulme S (2010). Subseafloor seawater-basalt-microbe reactions: continuous sampling of borehole fluids in a ridge flank environment. Geochem Geophys.

[20] Lin HT, Cowen JP, Olson EJ, Amend JP, Lilley MD (2012). Inorganic chemistry, gas compositions and dissolved organic carbon in fluids from sedimented young basaltic crust on the Juan de Fuca Ridge flanks. Geochim Cosmochim Acta.

[21] Elderfield H, Schultz A (1996). Mid-ocean ridge hydrothermal fluxes and the chemical composition of the ocean. Annu Rev Earth Planet Sci.

[22] Orcutt BN, Sylvan JB, Knab NJ, Edwards KJ (2011). Microbial ecology of the dark ocean above, at, and below the seafloor. Microbiol Mol Biol Rev.

[23] Jungbluth SP, Bowers RM, Lin H, Cowen JP, Rappé MS (2016). Novel microbial assemblages inhabiting crustal fluids within mid-ocean ridge flank subsurface basalt. ISME J.

[24] Jungbluth SP, Grote J, Lin H, Cowen JP, Rappé MS (2013). Microbial diversity within basement fluids of the sediment-buried Juan de Fuca Ridge flank. ISME J.

[25] Rinke C, Schwientek P, Sczyrba A, Ivanova NN, Anderson IJ, Cheng JF (2013). Insights into the phylogeny and coding potential of microbial dark matter. Nature.

[26] Parks DH, Chuvochina M, Waite DW, Rinke C, Skarshewski A, Chaumeil P-A. A proposal for a standardized bacterial taxonomy based on genome phylogeny. *Nat Biotechnol* 2018;36;996–1004. 10.1038/nbt.4229 30148503

[27] Fisher A.T., Wheat C.G., Becker K., Cowen J., Orcutt B., Hulme S., Inderbitzen K., Haddad A., Pettigrew T.L., Davis E.E., Jannasch H., Grigar K., Aduddell R., Meldrum R., Macdonald R., Edwards K.J. (2011). Design, deployment, and status of borehole observatory systems used for single-hole and cross-hole experiments, IODP Expedition 327, eastern flank of Juan de Fuca Ridge. Proceedings of the IODP.

[28] Orcutt B.N., Barco R.A., Joye S.B., Edwards K.J. (2012). Summary of carbon, nitrogen, and iron leaching characteristics and fluorescence properties of materials considered for subseafloor observatory assembly. Proceedings of the IODP.

[29] Orcutt B, Wheat CG, Edwards KJ (2010). Subseafloor ocean crust microbial observatories: development of FLOCS (FLow-through Osmo Colonization System) and evaluation of borehole construction materials. Geomicrobiol J.

[30] Stepanauskas R, Fergusson EA, Brown J, Poulton NJ, Tupper B, Labonté JM (2017). Improved genome recovery and integrated cell-size analyses of individual uncultured microbial cells and viral particles. Nat Commun.

[31] Chen IMA, Markowitz VM, Chu K, Palaniappan K, Szeto E, Pillay M (2017). IMG/M: integrated genome and metagenome comparative data analysis system. Nucleic Acids Res.

[32] Huntemann M, Ivanova NN, Mavromatis K, Tripp HJ, Paez-Espino D, Palaniappan K (2015). The standard operating procedure of the DOE-JGI Microbial Genome Annotation Pipeline (MGAP v.4). Stand Genom Sci.

[33] Alneberg J, Bjarnason BS, de Bruijn I, Schirmer M, Quick J, Ijaz UZ (2014). Binning metagenomic contigs by coverage and composition. Nat Meth.

[34] Eren AM, Esen OumlC, Quince C, Vineis JH, Morrison HG, Sogin ML (2015). Anvi’o: an advanced analysis and visualization platform for ‘omics data. PeerJ.

[35] Parks DH, Imelfort M, Skennerton CT, Hugenholtz P, Tyson GW (2015). CheckM: assessing the quality of microbial genomes recovered from isolates, single cells, and metagenomes. Genome Res.

[36] Stamatakis A (2014). RAxML Version 8: a tool for phylogenetic analysis and post-analysis of large phylogenies. Bioinformatics.

[37] Pruesse E, Peplies J, Glöckner FO (2012). SINA: accurate high-throughput multiple sequence alignment of ribosomal RNA genes. Bioinformatics.

[38] Schloss PD, Westcott SL, Ryabin T, Hall JR, Hartmann M, Hollister EB (2009). Introducing mothur: open-source, platform-independent, community-supported software for describing and comparing microbial communities. Appl Environ Microbiol.

[39] Edgar RC (2004). MUSCLE: multiple sequence alignment with high accuracy and high throughput. Nucleic Acids Res.

[40] Capella-Gutiérrez S, Silla-Martínez JM, Gabaldón T (2009). trimAl: a tool for automated alignment trimming in large-scale phylogenetic analyses. Bioinformatics.

[41] Olm MR, Brown CT, Brooks B, Banfield JF (2017). dRep: a tool for fast and accurate genomic comparisons that enables improved genome recovery from metagenomes through de-replication. ISME J.

[42] Price MN, Dehal PS, Arkin AP (2010). FastTree 2 – approximately maximum-likelihood trees for large alignments. PLOS ONE.

[43] Ludwig W, Strunk O, Westram R, Richter L, Meier H, Yadhukumar (2004). ARB: a software environment for sequence data. Nucleic Acids Res.

[44] Ver Eecke HC, Butterfield DA, Huber JA, Lilley MD, Olson EJ, Roe KK (2012). Hydrogen-limited growth of hyperthermophilic methanogens at deep-sea hydrothermal vents. Proc Natl Acad Sci.

[45] Luton PE, Wayne JM, Sharp RJ, Riley PW (2002). The mcrA gene as an alternative to 16S rRNA in the phylogenetic analysis of methanogen populations in landfill. Mircobiology.

[46] Giovannoni SJ, Tripp HJ, Givan S, Podar M, Vergin KL, Baptista D (2005). Genome streamlining in a cosmopolitan oceanic bacterium. Science.

[47] Baker BJ, Comolli LR, Dick GJ, Hauser LJ, Hyatt D, Dill BD (2010). Enigmatic, ultrasmall, uncultivated Archaea. Proc Natl Acad Sci. USA.

[48] Nakagawa S, Inagaki F, Suzuki Y, Steinsbu BO, Lever MA, Takai K (2006). Microbial community in black rust exposed to hot ridge flank crustal fluids. Appl Environ Microbiol.

[49] Hoshino T, Morono Y, Terada T, Imachi H, Ferdelman T, Inagaki F (2011). Comparative study of subseafloor microbial community structures in deeply buried coral fossils and sediment matrices from the challenger mound in the porcupine seabight. Front Microbiol.

[50] Foster PG, Cox CJ, Embley TM (2009). The primary divisions of life: a phylogenomic approach employing composition-heterogeneous methods. Philos Trans R Soc Lond B Biol Sci.

[51] Holm NG, Holm NG (1992). Why are hydrothermal systems proposed as plausible environments for the origin of life?. Marine Hydrothermal Systems and the Origin of Life.

[52] Anantharaman K, Jungbluth SP, Kantor R, Lavy A, Warren L, Rappé MS (2018). Expanded diversity of microbial groups that shape the dissimilatory sulfur cycle. ISME J.

[53] Hocking WP, Roalkvam I, Magnussen C, Stokke R, Steen IH (2015). Assessment of the carbon monoxide metabolism of the hyperthermophilic sulfate-reducing archaeon *Archaeoglobus fulgidus* VC-16 by comparative transcriptome analyses. Archaea.

[54] Robador A, Jungbluth SP, Larowe DE, Bowers RM, Rappé MS, Amend JP (2015). Activity and phylogenetic diversity of sulfate-reducing microorganisms in low-temperature subsurface fluids within the upper oceanic crust. Front Microbiol.

[55] Lever MA, Rouxel O, Alt JC, Shimizu N, Ono S, Coggon RM (2013). Evidence for microbial carbon and sulfur cycling in deeply buried ridge flank basalt. Science.

[56] Lau MCY, Cameron C, Magnabosco C, Brown CT, Schilkey F, Grim S (2014). Phylogeny and phylogeography of functional genes shared among seven terrestrial subsurface metagenomes reveal N-cycling and microbial evolutionary relationships. Front Microbiol.

[57] Kletzin A, Heimerl T, Flechsler J, van Niftrik L. Cytochromes c in Archaea: distribution, maturation, cell architecture, and the special case of Ignicoccus hospitalis. *Front Microbiol* 2015;6;1–15. 10.3389/fmicb.2015.00439 PMC4429474 26029183

[58] Adam Panagiotis S., Borrel Guillaume, Gribaldo Simonetta (2018). Evolutionary history of carbon monoxide dehydrogenase/acetyl-CoA synthase, one of the oldest enzymatic complexes. Proceedings of the National Academy of Sciences.

[59] Diender M, Stams AJM, Sousa DZ, Robb FT, Guiot SR (2015). Pathways and bioenergetics of anaerobic carbon monoxide fermentation. Front Microbiol.

[60] Fox JD, Kerby RL, Roberts GP, Ludden PW (1996). Characterization of the CO-induced, CO-tolerant hydrogenase from Rhodospirillum rubrum and the gene encoding the large subunit of the enzyme. J Bacteriol.

[61] Wu M, Ren Q, Durkin AS, Daugherty SC, Brinkac LM, Dodson RJ, et al. Life in hot carbon monoxide: the complete genome sequence of Carboxydothermus hydrogenoformans Z-2901. *PLoS ONE.* 2005;1:0563–0574. 10.1371/journal.pgen.0010065 PMC1287953 16311624

[62] Henstra AM, Dijkema C, Stams AJM (2007). *Archaeoglobus fulgidus* couples CO oxidation to sulfate reduction and acetogenesis with transient formate accumulation. Environ Microbiol.

[63] Techtmann SM, Colman AS, Robb FT (2009). Minireview: “That which does not kill us only makes us stronger”: the role of carbon monoxide in thermophilic microbial consortia. Environ Microbiol.

[64] Ragsdale SW (2004). Life with carbon monoxide. Crit Rev Biochem Mol.

[65] Stetter KO, Lauerer G, Thomm M, Neuner A (1987). Isolation of extremely thermophilic sulfate reducers: evidence for a novel branch of Archaebacteria. Science.

[66] Lazar CS, Baker BJ, Seitz KW, Teske AP (2017). Genomic reconstruction of multiple lineages of uncultured benthic archaea suggests distinct biogeochemical roles and ecological niches. ISME J.

[67] Mwirichia R, Intikhab A, Rashid M, Vinu M, Ba-Alawi W, Kamau AA (2016). Metabolic traits of an uncultured archaeal lineage -MSBL1- from brine pools of the Red Sea. Sci Rep.

[68] Weiss MC, Sousa FL, Mrnjavac N, Neukirchen S, Roettger M, Nelson-Sathi S, et al. The physiology and habitat of the last universal common ancestor. *Nat Microbiol*. 2016; 1:1-8. 10.1038/nmicrobiol.2016.116 27562259

[69] Hocking WP, Stokke R, Roalkvam I, Steen IH (2014). Identification of key components in the energy metabolism of the hyperthermophilic sulfate-reducing archaeon *Archaeoglobus fulgidus* by transcriptome analyses. Front Microbiol.

[70] Larowe DE, Dale AW, Amend JP, Van Cappellen P (2012). Thermodynamic limitations on microbially catalyzed reaction rates. Geochim Cosmochim Acta.

[71] Lin H, Cowen JP, Olson EJ, Lilley MD, Jungbluth SP, Wilson ST (2014). Dissolved hydrogen and methane in the oceanic basaltic biosphere. Earth Planet Sci Lett.

[72] Boettger J, Lin H, Cowen JP, Hentscher M, Amend JP (2013). Energy yields from chemolithotrophic metabolisms in igneous basement of the Juan de Fuca ridge flank system. Chem Geol.

[73] Canovas PA, Hoehler T, Shock EL (2017). Geochemical bioenergetics during low-temperature serpentinization: An example from the Samail ophiolite, Sultanate of Oman. J Geophys Res Biogeosci.

[74] Reeves EP, McDermott JM, Seewald JS (2014). The origin of methanethiol in midocean ridge hydrothermal fluids. Proc Natl Acad Sci USA.

[75] Lang K, Klingl A, Poehlein A, Daniel R, Brune A (2015). New mode of energy metabolism in the seventh order of methanogens as revealed by comparative genome analysis of “*Candidatus* Methanoplasma termitum”. Appl Environ Microbiol.

[76] Kono T, Mehrotra S, Endo C, Kizu N, Matusda M, Kimura H (2017). A RuBisCO-mediated carbon metabolic pathway in methanogenic archaea. Nat Commun.

[77] Meyer JL, Jaekel U, Tully BJ, Glazer BT, Wheat CG, Lin HT (2016). A distinct and active bacterial community in cold oxygenated fluids circulating beneath Mid-Atlantic seafloor. Sci Rep.

[78] van Wolferen M, Orell A, Albers SV (2018). Archaeal biofilm formation. Nat Rev Microbiol.

[79] Albers SV, Jarrell KF (2018). The archaellum: an update on the unique archaeal motility structure. Trends Microbiol.

[80] Hoehler TM, Jorgensen BB (2013). Microbial life under extreme energy limitation. Nat Rev Micro.

[81] Orsi WD, Edgcomb VP, Christman GD, Biddle JF (2013). Gene expression in the deep biosphere. Nature.

[82] Taylor JR, Stocker R (2012). Trade-offs of chemotactic foraging in turbulent water. Science.

[83] Bowers RM, Kyrpides NC, Stepanauskas R, Harmon-Smith M, Doud D, Reddy TBK (2017). Minimum information about a single amplified genome (MISAG) and a metagenome-assembled genome (MIMAG) of bacteria and archaea. Nat Biotechnol.

