# Supplementary material for: Carboxydotrophy potential of uncultivated Hydrothermarchaeota from the subseafloor crustal biosphere
Source: ISME J. 2019 Feb 7;13(6):1457–68. doi: 10.1038/s41396-019-0352-9 (PMC6775978; doi:10.1038/s41396-019-0352-9)
Supplement: Supplementary file 1 — Supplemental Material [file 41396_2019_352_MOESM1_ESM.docx]

Supplemental Information for *Carboxydotrophy potential of uncultivated Hydrothermarchaeota from the subseafloor crustal biosphere*

**Contributors:**

Stephanie A Carr^1, 2^, Sean P Jungbluth^3,4, 5^, Emiley A Eloe-Fadrosh^4^, Ramunas Stepanauskas^1^, Tanja Woyke^4^, Michael S Rappé^5*^, Beth N Orcutt^1*^

**Affiliations:**

(1) Bigelow Laboratory for Ocean Sciences, East Boothbay, ME, USA

(2) Hartwick College

(3) Center for Dark Energy Biosphere Investigations, University of Southern California, Los Angeles, CA, USA

(4) Department of Energy, Joint Genome Institute, Walnut Creek, CA, USA

(5) Hawaii Institute of Marine Biology, University of Hawaii at Manoa, Kaneohe, HI, USA

*Corresponding authors:

Beth Orcutt, 60 Bigelow Drive, East Boothbay, Maine, 04544, USA, Telephone: +1-207-315-2567; borcutt@bigelow.org; Michael Rappé, PO BOX 1346, Hawaii Institute of Marine Biology, Kaneohe, Hawaii, 96744, USA, Telephone: +1-808-236-7464; rappe@hawaii.edu

**Supplemental Information**

*Methods*

***Phylogenetic analyses***

16S rRNA genes were retrieved from dereplicated set of SAGs and MAGs and used to build the phylogenomic tree using the CheckM ssu_finder (Parks et al., 2015). Only sequences greater than 1000 bp were utilized. Because several closely related lineages (Theionarchaea, Hadesarchaea and MSBL1) lacked SAGs and MAGs with a 16S rRNA sequence greater than 1000 bps, additional sequences were retrieved from the SILVA rRNA database. Additional *Candidatus* Hydrothermarchaeota sequences were also extracted from the SILVA rRNA database. See Table S8 for accession numbers. All sequences were aligned using the SILVA Incremental Aligner (SINA) online tool (Pruesse et al., 2012). The alignment was masked out with the lane1349 mask using the *mothur* command filter.seqs (Schloss et al., 2009) and inspected manually in ARB (Wolfgang et al., 2004). The phylogenetic tree was constructed using raxmlHPC (RAxML version 8.2.8, Stamatakis, 2014) with the GTRCAT model and 1000 bootstrap replicates. Shorter Hydrothermarchaea rRNA sequences from this study (SAGs AC-334-K11 and AC-335-G1) were placed into the base tree using the parsimony tool in ARB (Ludwig et al., 2014).

A phylogenetic tree demonstrating the evolution of nitrate reductase genes was constructed using all the available the *napA* and *nasA* genes from IMG, and a few additional *nasA* genes and *fdh* (outgroup) from National Center for Biotechnology Information (NCBI) (Table S29). The napA genes were clustered using UCLUST at 75% amino acid similarity. Similarly, the evolutionary source of carbon-fixation pathways was evaluated with a non-rooted phylogenetic tree of the RuBisCO gene using 80 RuBisCO amino acid sequences curated by Kono et al. (2017, Table S30). For both phylogenies, sequences were aligned using MUSCLE (version 3.8.31, Edgar et al., 2004), trimmed and masked using trimAl (version 1.2rev59, flags: -gt 0.8 -st 0.001 -cons 60, Capella-Cutierrez et al., 2009), and manually inspected. The trees were constructed using raxMLHPC (verision 8.2.8) with the PROTGAMMAWAG model and 100 replicates.

***Amplification of mcrA gene***

Amplification of the *mcrA* gene, a marker for methane cycling potential in Archaea (Luton et al., 2002), was attempted using 20 µL reaction volumes with 10 µL of QIAGEN Fast Cycling PCR Master Mix (Qiagen, Cat. No. 203741, Hilden, Germany), 2 µl of DNA from MDA amplification of a single cell, 6 µL nuclease-free water and1 µL of each primer (forward qmcrA-alt 5´- GAR GAC CAC TTY GGH GGT TC- 3´ (Ver Eecke et al., 2012) and reverse ML 5´- TTC ATT GCR TAG TTW GGR TAG TT-3´ (Luton et al., 2002), each 20 pmol/µL). The cycling program was 95°C for 5 sec, followed by 35 cycles of 95°C for 5 sec, 59 °C for 5 sec, 68 °C for 15 sec, and a final extension at 72°C for 1 min. A mixture of two linearized *mcrA* plasmids (50:50 mixture generated from *Methanococcus jannaschii* and *Methanosarcina acetovorans*) was used as a positive control (Orcutt et al. 2015).

***Thermodynamic calculations of Gibbs Free Energy***

To determine the possible energetic yield of carbon monoxide oxidation coupled to sulfate reduction as compared to other possible electron donors, the Gibbs free energy was calculated for various reactions as shown in Table S17. The reactions were normalized to mole of electron donor (*i.e.,* hydrogen, methane, acetate, and carbon monoxide), and free energy yields were calculated both per mole of limiting electron donor as well as per mole of electron transferred for cross comparison between samples. The free energy yield of the reactions under *in situ* conditions (∆G_RXN,T_) was determined from Equation 1:

∆G_RXN,T_ = ∆Gº_RXN,T_ + *RT*ln(Q) (Equation S1)

where ∆Gº_RXN,T_ is the standard free energy yield of the reaction (in kJ mol^-1^) at 65ºC using free energy of formation values from Amend and Shock 2001 (Table S18), *R* is the universal gas constant (8.314 J K^–1^ mol^–1^), *T* is the absolute temperature (338 K), and Q is the ratio of the free energy of the formation of the species involved in the reaction according to Equation 2:

Q = ∑*(a*_i_^vi^, products)/(*a*_i_^vi^, reactants) (Equation S2)

where *a* is the activity of compound *i* and *v* is the stoichiometric coefficient. The activities were calculated from the activity coefficients assuming an ionic strength of 0.72 and *in situ* concentrations, as shown in Table S18.

Where possible, known *in situ* concentrations were used for the calculations (*i.e.*, for dissolved hydrogen and methane from (Lin et al., 2014); for sulfate, sulfide, ∑CO_2_, and pH from (Wheat et al., 2010). To our knowledge, carbon monoxide concentrations have not been measured in this system. Possible high-end values (up to 100 µM) were estimated from values of other deep-sea hydrothermal systems presented elsewhere (Reeves et al., 2014), and lower values were also included to determine thermodynamic feasibility at low concentrations (down to 10 nM). Similarly, to our knowledge, acetate concentrations have also not been determined for this system, so a similar estimated range of concentrations was used in the calculations (Table S18).

**Supplemental Figures**


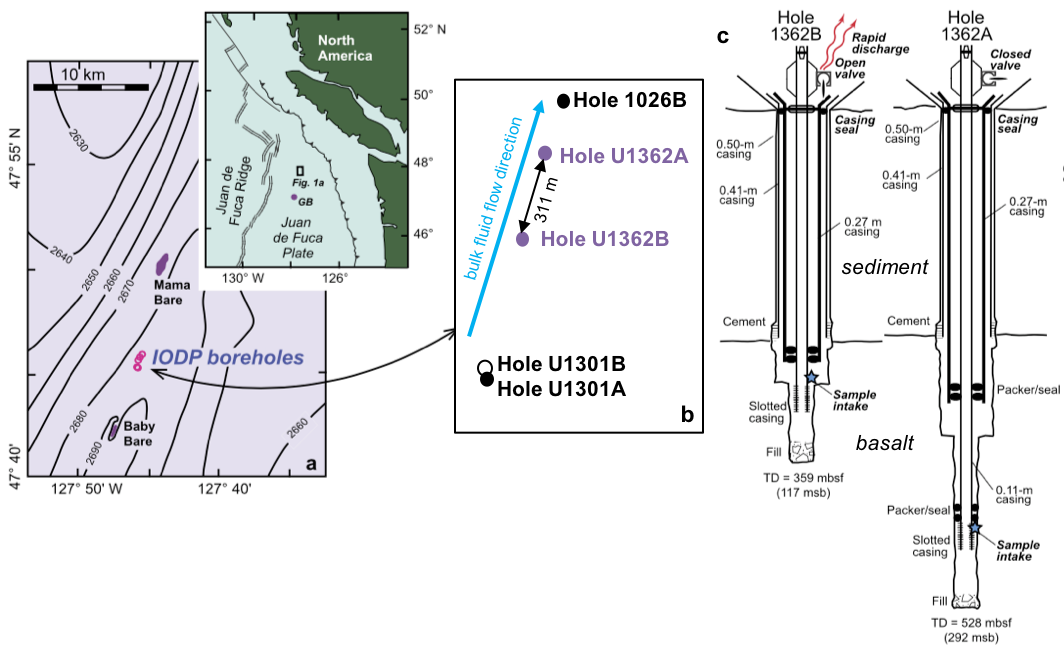


Figure S1. Juan de Fuca field site. (a) Location and bathymetry of the Juan de Fuca Ridge Flank ([Expedition 301 Scientists, 2005; Expedition 327 Scientists, 2011a; Shipboard Scientific Party, 1997](https://www.sciencedirect.com/science/article/pii/S0012821X16303351#br0310)). Inset index map shows location of Grizzly Bare outcrop (purple dot, “GB”)

(b) Orientation of borehole observatories in relation to the direction of crustal fluid flow. (c) Schematic of boreholes observatories at Holes 1362A and 1362B.

**Figure S2.** Locations where *Candidatus* Hydrothermarchaeota has been detected previously (see Table S1 for more information on the relatives taxa abundance and references).

**Figure S3**. Phylogenetic associations of the Juan de Fuca Ridge flank *Candidatus* Hydrothermarchaeota 16S rRNA gene sequences relative to other archaeal sequences. Black (100%) and white (99-80%) circles at nodes indicate branching bootstrap support values. For phylogenetic associates within *Ca.* Hydrothermarchaeota see Figure 1.

**Figure S4**. Metabolism overview of *Candidatus* Hydrothermarchaeota SAGs and MAGs from Juan de Fuca Ridge flank crustal subsurface, based on number of genes present in each genome. Gene name abbreviations: CO dehydrogenase/acetyl-CoA synthase (subunits alpha, A; epsilon, B; beta, C; delta, D; gamma, E), cdhABCDE; CO dehydrogenase maturation factor, cooC; carbon-monoxide dehydrogenase catalytic subunit, cooS; formylmethanofuran dehydrogenase (subunits A-G), fwdABCDEFG; formylmethanofuran-tetrahydromethanopterin formyltransferase, ftr; methenyltetrahydromethanopterin cyclohydrolase, mch; methylenatetrahydromethanopterin dehydrogenase, mtd; methylenetetrahydromethanopterin reductase, mer; tetrahydromethanopterin S-methyltransferase (subunits A), mtrA; CoB--CoM heterodisulfide reductase (subunits B-D), hdrBCD; formate dehydrogenase, fdo; cytochrome b, b-type; cytochrome b6, b6; Cytochrome C assembly protein; c assemb. Cytochrome c biogenesis protein, c biogen., cytochrome c oxidase, c oxidase; cytochrome c, c-type; Cytochrome c554, c554; cytochrome c7, Cbb3-type cytochrome oxidase, cbb3; Seven times multi-haem cytochrome CxxCH, multihaem; ATP-ase related pilus assembly protein, cpaF; twitching motility two-component system (subunits G,H), pilGH; chemotaxis response regulator (subunits B,Y) CheBY; chemotaxis protein (subunits A,C,D), cheACD, chemotaxis protein methyltransferase, cheR; methyl-accepting chemotaxis protein, mcp; purine-binding chemotaxis protein, cheW; Flagella accessory protein, flaC; flagellar protein (subunits G,H,I,J) FlaGHIJ; dissimilatory adenylylsulfate reductase (subunits A, B), apr; phosphoadenosine phosphosulfate reductase, cycH; sulfate adenylyltransferase subunit 2; cycD; sulfite reductase alpha (subunits, A, B), dsrAB; sulfate adenylyltransferase, sat; sulfate permease, sulP; sulfur relay complex (subunits C) dsrC; putative sulfite reductase-associated electron transfer protein, (subunits K,M) dsrKM; nitrate reductase (subunits ADGH), NapADGH; nitrate reductase (subunit alpha), narG; nitrate reductase (subunit gamma), narI; respiratory nitrate reductase chaperone, NarJ; nitrate/nitrite transporter, narK; hydrogenase nickel incorporation protein (subunits A-F), hypABCDEF; NiFe hydrogenase subunits (large or small), NiFe-Hases; coenzyme F420 hydrogenase subunit beta, frhB; 3-hexulose-6-phosphate synthase, hps; 6-phospho-3-hexuloisomerase, phi; fructose-bisphosphate aldolase, fba; phosphoglycerate kinase, pgk; ribulose-bisphosphate carboxylase, RuBisCO; triosephosphate isomerase, tpi.

­­

**Figure S5.** (A) Phylogenetic tree demonstrating the evolution of the assimilatory form of nitrate reductase (nasA, represented by squares) to the periplasmic form (napA, represented by circles). Hydrothermarchaeota are highlighted in brown. Accession numbers for each branch can be found in Table S29. Black (100%) and white (99-50%) circles indicate nodes with high local support values, from 100 replicates. The *napA* gene is thought to have evolved from the nitrate assimilation gene nasA (Stolz and Basu, 2002). To date, the earliest-evolved forms of the *napA* gene are monomeric (lacking napB), and can be found in bacteria such as *Desulfovibrio desulfuricans* and *Symbiobacterium thermophilum* (Jepson et al., 2006). Analysis of a *nasA*/*napA* phylogenetic tree suggests that *Candidatus* Hydrothermarchaota *napA* genes are a basal, early evolving lineage compared to those previously identified as *napA*. Similar to *D. desulfuricans* and *S. thermophilim,* the *napA* gene co-located with *napGHD* subunits. *narHG* genes are putative ferredoxins and are predicted to pass electrons from the quinone pool to napA via the c-type cytochrome napC or napM (Watkins et al., 2014). The *Ca.* Hydrothermarchaeota operon lacks *napC* and *napM*, but is co-located with a gene for a transmembrane cytochrome c-7. Coincidently, *Carboxydothermus ferrireducens*, a bacterial carboxydotroph, also contains an early-evolved *napA* gene. Given that the evolutionary progression of *nasA* to *napA* does not follow vertical inheritance, horizontal gene transfer probably contributed to the distribution of nitrate reduction in the Hydrothermarchaeota genomes.


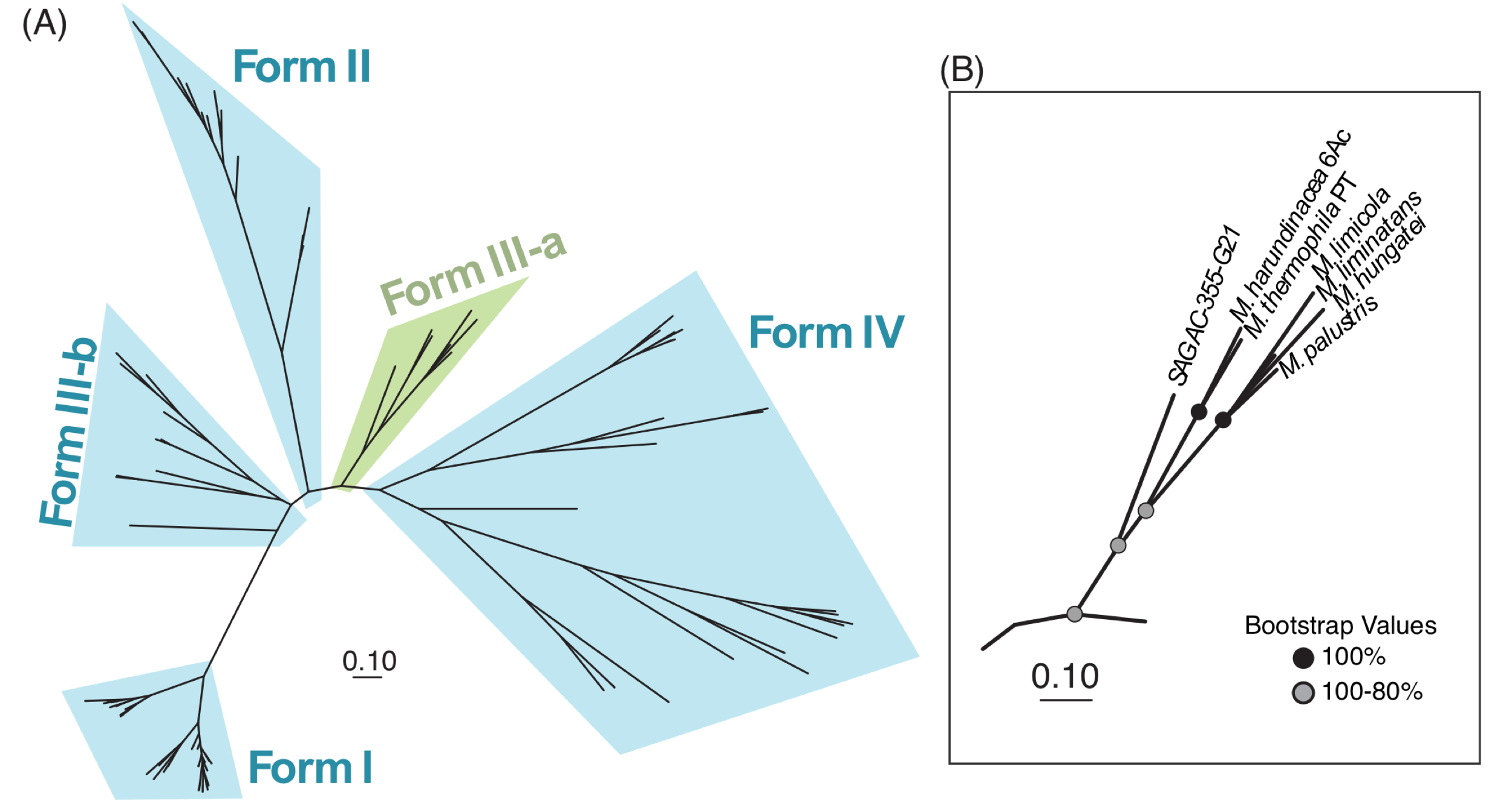


**Figure S6.** (A) Phylogenetic tree of RuBisCO and RuBisCO-like proteins. The co-assembled *Candidatus* Hydrothermarchaeota bin contained two genes that group within archaeal form III-a RuBisCO genes (Table S30), suggesting that *Ca.* Hydrothermarchaeota uses the reductive hexulose-phosphate pathway to fix CO_2_ for the production of a variety of metabolites. (B) Phylogenetic relationships of group Form III-a.

Supplemental Tables

**Table S1 (SEE EXCEL FILE).** Locations where *Candidatus* Hydrothermarchaeota has been previously detected by 16S rRNA gene sequencing, relative abundance of *Ca.* Hydrothermarchaeota (Hydrotherm) 16S rRNA genes within the sequence library, and *in situ* temperature (temp) and methane presence. Relative abundances are reported as the percentages of the sequenced archaeal communities when archaeal-specific primers were utilized and cannot be compared to bacterial abundances. The exception is Todorov et al., (2006) which used universal primers.

**Table S2**. Summary of fluid samples collected from the Juan de Fuca Ridge flank observatories during R/V *Atlantis* cruise AT18-07 and associated Hydrothermarchaeota single amplified genomes (SAGs) and metagenome-assembled genomes (MAGs).

| IODP Hole | **U1362A** | **U1362B** |
| --- | --- | --- |
| Latitude (N) | 47º45.662’ | 47º45.499’ |
| Longitude (W) | 127º45.674’ | 127º45.733’ |
| ROV Jason Dive # | J2-573 | J2-569 |
| Collection date | July 12, 2011 | July 8, 2011 |
| Water depth (m) | 2658 | 2658 |
| Fluid origin basement interval (mbsf) | 200-280 | 30-110 |
| Hydrothermarchaeota SAG IDs | AC-708-L17, AC-708-N22 | AC-334-K11,  AC-335-G21,  AC-335-L21 |
| Metagenome sample IDs | SSF21-22 | SSF23-24 |
| Raw Metagenome Taxon IDs in IMG | 3300002481 | 3300002532 |
| Metagenome Gold Analysis Project ID | Ga0004278 | Ga0004277 |
| NCBI Biosample accession | SAMN03166137 | SAMN03166138 |
| Hydrothermarchaeota MAG IDs | JdFR-17,  JdFR-18 | JdFR-16 |

**Table S3.** Taxon accession numbers for single amplified genomes and metagenome-assembled genomes within the Integrated Microbial Genomes & Microbiomes (IMG/M) platform.

| Sample Name | IMG accession number | NCBI Accession |
| --- | --- | --- |
| AC-334-K11 | 2522125056 | Taxonomy ID: 74985 |
| AC-335-G21 | 2634166800 | SAMN02744672 |
| AC-335-L21 | 2634166805 | SAMN02744675 |
| AC-708-L17 | 2634166822 | SAMN02744590 |
| AC-708-N22 | 2634166825 | SAMN02744594 |
| JdFR-16 | 2728369317 | SAMN03166138 |
| JdFR-17 | 2728369322 | SAMN03166137 |
| JdFR-18 | 2728369320 | SAMN03166137 |

**Table S4 (SEE EXCEL FILE).** Names, accession numbers, and CheckM statistics (Parks et al., 2015) of genomes used to build the phylogenomic tree shown in Figure 1b.

**Table S5.** **(SEE EXCEL FILE)** Single copy marker genes used for the concatenated alignment and phylogenomic tree shown in Figure 1b.

**Table S6.** Comparison of the 16S rRNA gene sequence identity (from 0-1, with 0 representing no sequence overlap) and sequence alignment fraction (align fract., as a percent) between *Candidatus* Hydrothermarchaeota single amplified genomes (SAGs) and metagenome-assembled genomes (MAGs).

|  |  | Genomes | | | | | | | | |
| --- | --- | --- | --- | --- | --- | --- | --- | --- | --- | --- |
|  |  | SAGs | | | | | | MAGs | | |
| Query |  | AC-335-L21 | AC-708-L17 | AC-335-G21 | AC-334-K11 | AC-708-N22 | JdFR-17 | | JdFR-18 |  |
| AC-335-L21 | identity | - | 0.10 | 0.99 | 0.99 | 0.99 | 0.99 | | 0.88 |  |
|  | align fract. (%) | - | 100 | 34 | 35 | 97 | 72 | | 100 |  |
| AC-708-L17 | Identity | 0.10 | - | 0.99 | 0.99 | 0.99 | 0.99 | | 0.88 |  |
|  | align fract. (%) | 100 | - | 34 | 35 | 97 | 72 | | 100 |  |
| AC-335-G21 | Identity | 0.99 | 0.99 | - | 0.99 | 0.99 | 0.99 | | 0.88 |  |
|  | align fract. (%) | 99 | 99 | - | 97 | 99 | 99 | | 97 |  |
| AC-334-K11 | Identity | 0.99 | 0.99 | 0.87 | - | 0.99 | 0.99 | | 0.87 |  |
|  | align fract. (%) | 99 | 100 | 100 | - | 100 | 100 | | 100 |  |
| AC-708-N22 | Identity | 0.99 | 0.99 | 0.99 | 0.99 | - | 0.99 | | 0.88 |  |
|  | align fract. (%) | 100 | 100 | 35 | 36 | - | 75 | | 100 |  |
| JdFR-17 | Identity | 0.99 | 0.99 | 0.99 | 0.99 | 0.99 | - | | 0.89 |  |
|  | align fract. (%) | 100 | 100 | 47 | 48 | 100 | - | | 98 |  |
| JdFR-18 | Identity | 0.88 | 0.88 | 0.88 | 0.87 | 0.88 | 0.98 | | - |  |
|  | align fract. (%) | 100 | 100 | 34 | 35 | 97 | 71 | | - |  |

**Table S7**. Average Nucleotide Identity (ANI) values and the number of bidirectional best hits (BBH) between *Candidatus* Hydrothermarchaeota SAGs and MAGs.

|  | Group A Genomes | | | | | | | | | | | | | | | | | |
| --- | --- | --- | --- | --- | --- | --- | --- | --- | --- | --- | --- | --- | --- | --- | --- | --- | --- | --- |
|  | MAG  JdFR-16 | | | MAG  JdFR-17 | | MAG  JdFR-18 | | SAG  AC-334-K11 | | SAG  AC-335-G21 | | SAG  AC-335-L21 | | | SAG  AC-708-L17 | | SAG  AC-708-N22 | |
| Group B Genomes | ANI | BBH | ANI | | BBH | ANI | BBH | ANI | BBH | ANI | BBH | ANI | BBH | ANI | | BBH | ANI | BBH |
| MAG JdFR-16 | - | - | 94.3 | | 1108 | 67.2 | 59 | 92.5 | 509 | 94.5 | 391 | 93.2 | 415 | 93.0 | | 632 | 92.7 | 228 |
| MAG JdFR-17 | 94.3 | 898 | - | | - | 67.3 | 104 | 96.7 | 739 | 96.0 | 521 | 96.1 | 574 | 97.4 | | 999 | 98.2 | 374 |
| MAG JdFR-18 | 67.4 | 63 | 67.6 | | 113 | - | - | 66.6 | 72 | 67.1 | 38 | 68.5 | 51 | 68.1 | | 96 | 68.6 | 34 |
| SAG AC-334-K11 | 92.4 | 512 | 96.8 | | 768 | 66.6 | 71 | - | - | 99.5 | 427 | 99.1 | 338 | 94.4 | | 648 | 89.9 | 214 |
| SAG AC-335-G21 | 94.4 | 400 | 96.2 | | 553 | 67.1 | 39 | 99.5 | 428 | - | - | 99.8 | 339 | 98.6 | | 428 | 89.8 | 69 |
| SAG AC-335-L21 | 93.0 | 420 | 96.2 | | 608 | 68.5 | 51 | 99.1 | 346 | 99.8 | 399 | - | - | 98.5 | | 693 | 90.9 | 228 |
| SAG AC-708-L17 | 92.9 | 640 | 97.5 | | 1041 | 68.2 | 96 | 98.4 | 656 | 98.5 | 428 | 98.5 | 693 | - | | - | 91.1 | 383 |
| SAG AC-708-N22 | 92.7 | 229 | 98.2 | | 337 | 68.6 | 34 | 89.7 | 221 | 89.8 | 69 | 91.0 | 228 | 91.1 | | 383 | - | - |

**Table S8 (SEE EXCEL FILE)**. Accession numbers of 16S rRNA genes used to make the 16S rRNA phylogenetic tree shown in Figure 1a

**Table S9** **(SEE EXCEL FILE)**. Genes identified within *Candidatus* Hydrothermarchaeota related to sulfate reduction.

**Table S10 (SEE EXCEL FILE)**. Genes identified within *Candidatus* Hydrothermarchaeota related to the reduction of nitrogen compounds.

**Table S11 (SEE EXCEL FILE)**. Genes identified within *Candidatus* Hydrothermarchaeota related to cytochromes.

**Table S12** **(SEE EXCEL FILE)**. Genes identified within *Candidatus* Hydrothermarchaeota related to carboxydotrophy.

**Table S13** **(SEE EXCEL FILE)**. Genes identified within *Candidatus* Hydrothermarchaeota related to the Wood-Ljungdahl pathway.

**Table S14** **(SEE EXCEL FILE)**. Genes identified within *Candidatus* Hydrothermarchaeota related to electron transfer.

**Table S15 (SEE EXCEL FILE).** Genes identified within *Candidatus* Hydrothermarchaeota related to methyamide transferase

**Table S16 (SEE EXCEL FILE).** Genes identified within *Candidatus* Hydrothermarchaeota related to hydrogenases.

**Table S17**. Potential reactions, the corresponding standard free energy yield of the reactions (∆G˚_RXN,T_; normalized per mole of limiting electron donor) at the *in situ* temperature of 65ºC (taken from data in Amend and Shock 2001), and the number of electron transferred in the reaction.

| **Balanced Reaction** | **∆Gº_RXN,T_ (kJ mol^–1^)** | ***e*^–^ transferred** |
| --- | --- | --- |
| H_2_(*aq*) + 0.25SO_4_^2–^ + 0.25H^+^ → 0.25HS^–^ + H_2_O | -65.9 | 2 |
| CH_4_(aq) + SO_4_^2–^ → HCO_3_^–^ + HS^–^ + H_2_O | -35.5 | 8 |
| CH_3_COO^–^ + SO_4_^2–^ → 2HCO_3_^–^ + HS^–^ | -55.5 | 8 |
| CO(*aq*) + 0.25SO_4_^2–^ + 0.25H^+^ → CO_2_(*aq*) + 0.25HS^–^ | -77.3 | 2 |

**Table S18**. Properties of compounds considered in the thermodynamic calculations.

| **Compound** | **∆G_f_ (kJ mol^–1^)^a^** | **Activity coefficient^b^** | **Concentration (µM)** |
| --- | --- | --- | --- |
| CH_4_ (*aq*) | -38.83 | 1 | 1.5 – 32^c^ |
| H_2_ (*aq*) | 14.88 | 1.15 | 0.05 – 2^c^ |
| CH_3_COO^–^ | -369.4 | 0.642 | 0.01 – 100 |
| CO (*aq*) | -125.0 | 1 | 0.01 – 100 |
| SO_4_^2–^ | -744.6 | 0.152 | 17,600^d^ |
| HS^–^ | 12.1 | 0.604 | 0.1^d^ |
| CO_2_ (*aq*) | -391.44 | 1 | 3.84^e^ |
| H_2_O | -240.24 | n.a. | n.a. |

a: Gibbs free energy of formation values calculated from Amend and Shock, 2001

b: Calculated assuming an ionic strength of 0.72

c: Range reported for Juan de Fuca Ridge flank CORK borehole fluids from Lin et al., 2014

d: Values from Wheat et al., 2010

e: Estimated from alkalinity (0.41 mM HCO_3_^–^) and pH (7.4) values reported in Wheat et al. 2010

**Table S19**. Free energy yield of carbon monoxide oxidation coupled to sulfate reduction in the Juan de Fuca Ridge flank crustal subsurface as compared to sulfate reduction with other electron donors.

| **Concentration (µM)** | **∆G_RXN,T_ (kJ mol^–1^)^a^** | **∆G_RXN,T_**  **(kJ mol^–1^ e^–1^)^a^** | **Source** | |
| --- | --- | --- | --- | --- |
| *Hydrogen* | | | |  |
| 0.05 | -11 | -5.5 | U1362A-2011^c^ | |
| 1.7 | -21 | -10.4 | U1362A-2013^c^ | |
| 0.08 | -12 | -6.1 | U1362B-2011^c^ | |
| 0.07 | -12 | -5.9 | U1362B-2013^c^ | |
| 0.3 | -16 | -8.0 | U1301A-2010^c^ | |
| 2 | -21 | -10.7 | U1301A-2008^c^ | |
| 0.7 | -18 | -9.2 | 1026B-2008^c^ | |
| *Methane* | | | |  |
| 6.1 | -55 | -6.9 | U1362A-2011^c^ | |
| 32 | -60 | -7.5 | U1362A-2013^c^ | |
| 13 | -57 | -7.1 | U1362B-2011^c^ | |
| 5 | -54 | -6.8 | U1362B-2013^c^ | |
| 1.6 | -51 | -6.4 | U1301A-2010^c^ | |
| 1.5 | -51 | -6.4 | U1301A-2008^c^ | |
| 2 | -52 | -6.5 | 1026B-2008^c^ | |
| *Acetate* | | | |  |
| 0.01 | -78 | -9.7 |  | |
| 0.1 | -84 | -10.5 |  | |
| 1 | -91 | -11.3 |  | |
| 10 | -97 | -12.1 |  | |
| 100 | -103 | -12.9 |  | |
| *Carbon Monoxide* | | | |  |
| 0.01 | -52 | -26.2 |  | |
| 0.1 | -59 | -29.4 |  | |
| 1 | -65 | -32.7 | EPR high temp. max^d^ | |
| 10 | -72 | -35.9 | Rainbow high temp. max^d^ | |
| 100 | -78 | -39.1 | Guaymas high temp. max^d^ | |

a: Calculated per mole of limiting electron donor

b: energy per mole of electron transferred

c: From Lin et al., 2014

d: From Reeves et al., 201

Table S20 **(SEE EXCEL FILE)**. Genes identified within *Candidatus* Hydrothermarchaeota related to sugar metabolism

Table S21 **(SEE EXCEL FILE)**. Genes identified within *Candidatus* Hydrothermarchaeota related to the incomplete citric acid cycle

Table S22 **(SEE EXCEL FILE)**. Genes identified within *Candidatus* Hydrothermarchaeota related to amino acid biosynthesis

Table S23 **(SEE EXCEL FILE)**. Genes identified within *Candidatus* Hydrothermarchaeota related to lipid biosynthesis

Table S24 **(SEE EXCEL FILE)**. Genes identified within *Candidatus* Hydrothermarchaeota related transporters

Table S25 **(SEE EXCEL FILE)**. Genes identified within *Candidatus* Hydrothermarchaeota related the reductive hexulose-phosphate cycle

Table S26 **(SEE EXCEL FILE)**. Genes identified within *Candidatus* Hydrothermarchaeota related to motility

Table S27 **(SEE EXCEL FILE)** Names, accession numbers, relative abundance of motility genes, and CheckM statistics (Parks et al., 2015) of genomes used to build Figure 4a.

Table S28 **(SEE EXCEL FILE)** Names, accession numbers, and relative abundance of motility genes of metagenomes used to build Figure 4b.

Table S29 **(SEE EXCEL FILE).** Name and accession numbers (IMG and NCBI) of genes used to build the napA/nasA phylogenomic tree shown in Figure S5. Figure IDs correspond to the numbered branches in Figure S5.

Table S30 **(SEE EXCEL FILE)**. Accession numbers of RuBisCO genes, and the name of the RuBisCO containing organism used to make the RuBisCO tree shown in Figure S6.

Supplemental References

Amend JP, Shock EL. Energetics of overall metabolic reactions of thermophilic and hyperthermophilic Archaea and Bacteria. *FEMS Microbiol Rev* 2001; 25:175–243.

Bowers RM, Kyrpides NC, Stepanauskas R, Harmon-Smith, M, Doud D, Jarett J, et al. Minimum information about a single amplified genome (MISAG) and a metagenome-assembled genome (MIMAG) of Bacteria and Archaea. *Nat Biotech* 2017; **35**(8).

Fisher AT, Urabe T, Klaus A, the Expedition 301 Scientists. IODP Expedition 301 Installs Three Borehole Crustal Observatories, Prepares for Three-Dimensional, Cross-Hole Experiments in the Northeastern Pacific Ocean. *Scientific Drilling* 2005; **1**: 6–11.

Fisher AT, Wheat CG, Becker K, Cowen J, Orcutt B, Hulme S, et al. Design, deployment, and status of borehole observatory systems used for single-hole and cross-hole experiments, IODP Expedition 327, eastern flank of Juan de Fuca Ridge. In Fisher AT, Tsuji T, Petronotis K, and the Expedition 327 Scientists (eds.), *Proc. IODP, 327*. Tokyo: Integrated Ocean Drilling Program Management International, Inc. 2001.

Jepson BJN, Marietou A, Mohan S, Cole JA, Butler CS, Richardson DJ. Evolution of the soluble nitrate reductase: defining the monomeric periplasmic nitrate reductase subgroup. *Biochemical Society Transactions* 2006; **34**(1), 122 -126.

Lin H-T, Cowen JP, Olson EJ, Lilley MD, Jungbluth SP, Wilson ST, et al. Dissolved methane and hydrogen in the oceanic basaltic biosphere. Earth and Planetary Science Letters 2014; **405**: 62-73.

Orcutt BN, Sylvan JB, Rogers DR, Delaney J, Lee RW, Girguis PR. Carbon fixation by basalt-hosted microbial communities. *Front Microbiol* (2015); **6**: 904.

Shipboard Scientific Party. Rough Basement Transect (Sites 1026 and 1027) Davis EE, Fisher AT, Firth J (eds.). *Proc ODP Initial Reports 168*, Ocean Drilling Program: College Station, TX, 1997, pp. 101-160.

Reeves EP, McDermott JM, Seewald JS. The origin of methanethiol in midocean ridge hydrothermal fluids. *Proc Natl Acad Sci* 2014; **111**(15): 5474-5479.

Wheat CG, Jannasch HW, Fisher AT, Becker K, Sharkey J, Hulme S. Subseafloor seawater-basalt-microbe reactions: Continuous sampling of borehole fluids in a ridge flank environment. *Geochemistry, Geophysics, Geosystems* 2010; **11**(7): 1–18.
